# Posterior Sinuplasty: A New Strategy for Managing Hydrocolpos in Cloaca—Case Series

**DOI:** 10.1055/a-2204-8629

**Published:** 2024-02-13

**Authors:** Amr AbdelHamid AbouZeid, Ahmed Saad Abdelmoniem, Mohamed Abdelrahman Fathy, Mohamed Ahmed Negm, Shaimaa Abdelsattar Mohammad

**Affiliations:** 1 Department of Pediatric Surgery, Ain Shams University Faculty of Medicine, Cairo, Egypt; 2 Department of Pediatric Surgery, Benha Health Insurance Hospital, Benha, Egypt; 3 Department of Pediatric Surgery, Minia University, El Minia, Egypt; 4 Department of Pediatric Surgery, South Valley University Qena Faculty of Medicine, Qena, Egypt; 5 Department of Radiodiagnosis, Ain Shams University Faculty of Medicine, Cairo, Egypt

**Keywords:** anorectal malformations, urogenital sinus, vaginal septum, Mullerian anomalies, congenital

## Abstract

We present a simple surgical technique aiming to improve urine outflow through the common urogenital sinus in cloaca and facilitate drainage of existing hydrocolpos. The study included three cases of cloaca with associated hydrocolpos that were operated during the period 2022 through 2023. The patient is placed in the prone position for a standard posterior sagittal anorectoplasty. The distal rectal fistula is severed flush with the vagina/sinus leaving an open defect in the posterior wall of the vagina/sinus. The defect is then widened distally via a vertical incision (∼1 cm) through the posterior wall of the common urogenital sinus toward but not reaching the perineum. This vertical defect is then closed horizontally displacing the posterior vaginal wall downwards toward the perineum (posterior sinuplasty). The postoperative recovery was uneventful in the three cases. Adequate drainage of hydrocolpos was confirmed by imaging at follow-up, as well as improvement of upper urinary tract dilatation. In selected cases of cloaca, posterior sinuplasty is a simple procedure that can be applied during anorectoplasty to provide effective drainage of associated hydrocolpos.

## Introduction

Cloaca is a complex anomaly in the female where the rectum, vagina, and urethra join to drain through a single perineal orifice. 1 Despite recent advances in the management of different types of anorectal anomalies, cloacal repair remains a challenge with less satisfactory functional outcomes even in best centers all over the world. 2 3 4 5

Management of the anorectal component in cloaca is relatively easier and straightforward compared with the urogenital part of the anomaly. Unlike the rectum, separation and mobilization of the vagina is not an easy job which is more liable for strictures and failure. 3 6 Some authors advocate delaying vaginal surgery after puberty when the vagina becomes “more forgiving” under the effect of hormonal stimulation. 5 A major limitation for delaying vaginal surgery in cloaca would be the presence of hydrocolpos, which is prevalent in 30 to 40% of cloacas. 4 7 8 The abnormal high urogenital confluence (common urogenital sinus) preferentially directs the urine into the vagina rather than to the outside. When the vagina becomes distended with urine (hydrocolpos), it compresses the trigone of the urinary bladder embedding its emptying and putting the upper urinary tract at risk (hydroureteronephrosis). 4 7 8

In this report, we present a simple surgical technique aiming to improve urine outflow through the common urogenital sinus in cloaca and facilitate drainage of existing hydrocolpos. The technique can be applied during anorectoplasty via the standard posterior sagittal approach.

## Case Report




**Video 1**


The study included three cases of cloaca that were operated during the period 2022 through 2023. The three cases underwent pelvic colostomy at birth before referral for the definitive repair. All cases had associated hydrocolpos; however, only one case underwent vaginostomy at the time of colostomy.

**Fig. 1:**
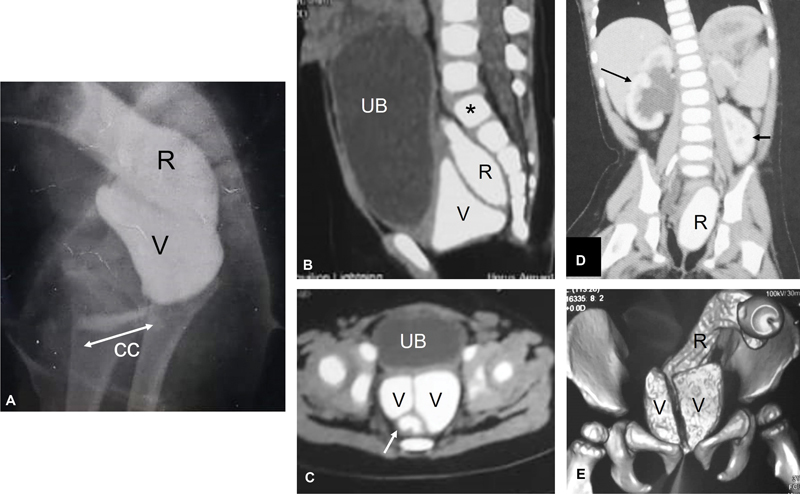
First case: Eighteen-month-old girl with cloaca. ( **A** ) Contrast X-ray study demonstrating the rectum (R), vagina (V), and common channel (CC). Computed tomography (CT) scan of abdomen and pelvis following contrast injection in vagina and rectum: sagittal, axial, coronal, and three-dimensional (3D) reconstruction ( **B** – **E** , respectively). Urinary bladder (UB); hemi-vagina (V); rectum (R); first sacral vertebra (*). Note: the presence of longitudinal vaginal septum between 2 hemi-vaginas ( **C** and **E** ); hydronephrotic right kidney (long black arrow in **D** ), and ectopic (low position) left kidney (short black arrow in **D** ).

**Fig. 2:**
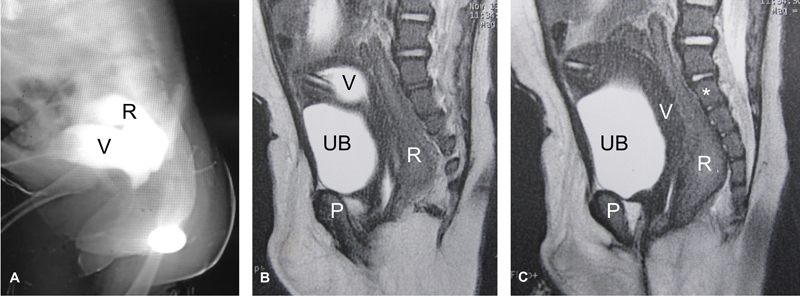
Second case: Seven-month-old girl with cloaca. ( **A** ) Contrast X-ray study demonstrating the rectum (R) and vagina (V). ( **B** and **C** ) Pelvic magnetic resonance imaging (MRI) (sagittal T2-weighted image [T2WI]) demonstrating urinary bladder (UB), vagina (v), rectum (R), pubis (P), and first sacral vertebra (*). Note the presence of tube in the vagina (vaginostomy tube).

**Fig. 3:**
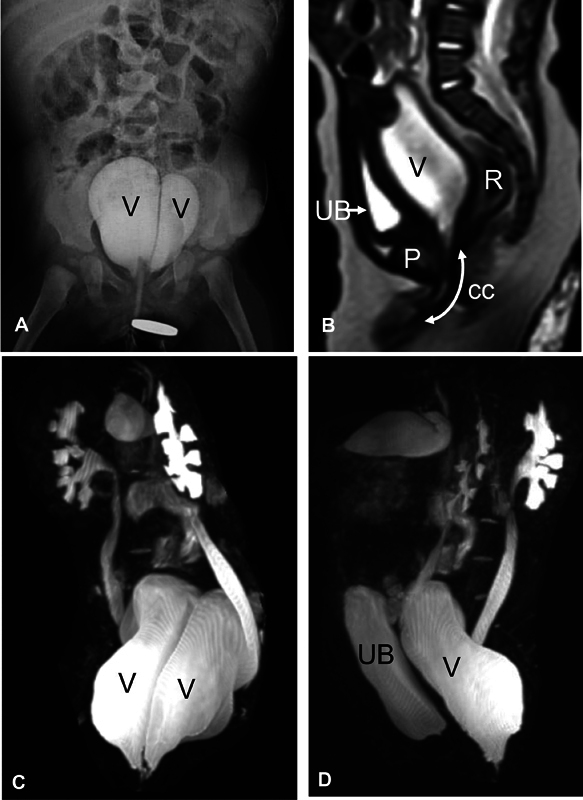
Third case: Six-month-old girl with cloaca. ( **A** ) Contrast X-ray study (genitogram) demonstrating two hemi-vaginas (V). ( **B** ) Pelvic magnetic resonance imaging (MRI) (sagittal T2-weighted image [T2WI]) demonstrating urinary bladder (UB), vagina (v), rectum (R), pubis (P), and common channel (cc). ( **C** and **D** ) Three-dimensional (3D) MR urography (coronal and sagittal, respectively) demonstrating bilateral hydroureteronephrosis, distended two hemi-vaginas (V), and distended urinary bladder (UB).

Preoperative imaging included conventional contrast X-ray studies (genitogram, distal loopogram) in all cases. Cross-sectional imaging studies were also performed: pelvic computed tomography scan in one ( Fig. 1 ) and pelvic magnetic resonance imaging (MRI) in the other two cases ( Figs. 2 and 3 ). In the three cases, the distal rectum was located opposite the fourth sacral vertebra (S4), which would favor a perineal approach for the anorectoplasty. 7 As regards the urogenital component of the anomaly, the lower end of the vagina was opposite the middle portion of the pubic symphysis in the three cases, which indicate an intermediate type (length of common channel ranged between 2 and 3 cm). 7 Longitudinal vaginal septum was present in two cases (Mullerian duplication anomalies) ( Figs. 1 and 3 ).

*Surgical technique* : We start by endoscopic examination of the common urogenital sinus (vaginoscopy, cystoscopy) to confirm imaging findings. A self-retaining Foley's catheter is placed over a guidewire and secured inside the urinary bladder at the end of endoscopy.

**Fig. 4:**
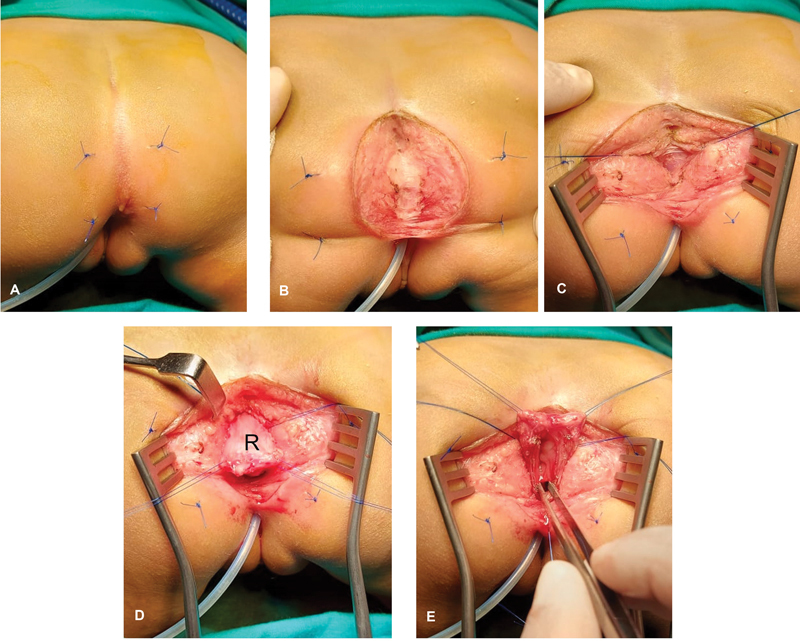
Operative steps of posterior sagittal anorectoplasty (PSARP). ( **A** ) The patient is positioned in prone position. ( **B** ) Midline posterior sagittal incision. ( **C** ) The levator ani is incised in the midline to reach the rectum above. ( **D** ) The rectum (R) is mobilized by dissection of perirectal fascia. ( **E** ) A vertical midline incision is made in the posterior wall of the mobilized distal rectum to expose its fistulous communication anteriorly into the vagina/sinus.

**Fig. 5:**
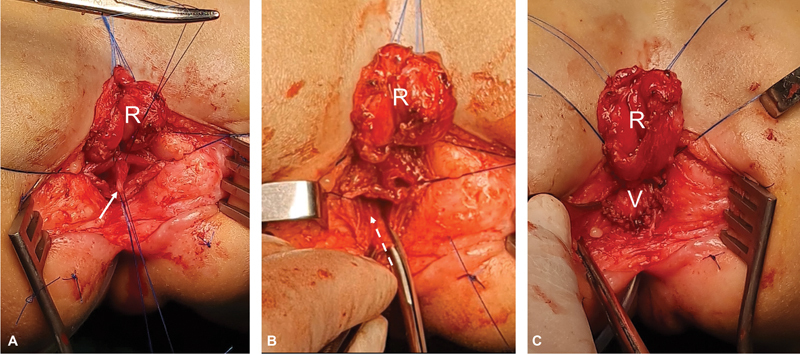
Operative steps of posterior sinuplasty. ( **A** ) Separation of the rectum (R) from the vagina to be followed by excision of longitudinal septum (white arrow). ( **B** ) A vertical incision (broken arrow) is made through the posterior wall of the common urogenital sinus toward but not reaching the perineum. ( **C** ) This vertical defect is then closed horizontally displacing the posterior vaginal wall (V) downwards toward the perineum.

The patient is then turned to the prone position for the posterior sagittal anorectoplasty (PSARP). 9 The predestined site of the normal anus is identified and marked on the skin by four stitches ( Fig. 4A ). A midline posterior sagittal incision is made from the coccyx down to but not exceeding the anterior margin of the anal sphincter. The incision is deepened in the midline splitting the vertical fibers of the striated sphincter complex anteriorly, while reaching deep to the diaphragmatic portion of the levator ani posteriorly ( Fig. 4B ). The levator ani is incised in the midline to reach the rectum above ( Fig. 4C ). The rectum is mobilized by dissection of perirectal fascia close to the rectal wall to avoid injury to pelvic nerves and adjacent structures ( Fig. 4D ). We start by the dissection on the posterior and lateral aspects of the rectum (suprafistula dissection) Video 1 . A vertical midline incision is made in the posterior wall of the mobilized distal rectum to expose its fistulous communication anteriorly into the vagina/sinus ( Fig. 4E ). Then, we proceed with the more challenging anterior separation of the rectum from the adherent posterior vaginal wall ( Fig. 5 ). The distal rectal fistula is severed flush with the vagina/sinus leaving an open defect in the posterior wall of the vagina/sinus. After separation of the anterior rectal wall from the vagina, we continue with rectal dissection and mobilization till it can reach the perineum without considerable tension.

**Fig. 6:**
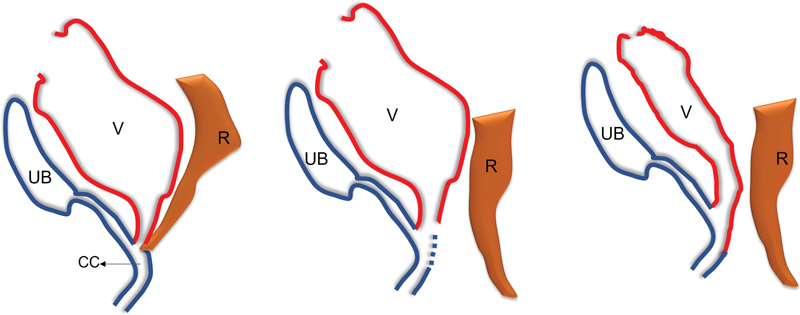
Diagram illustrating posterior sinuplasty. ( **A** ) A case of cloaca: Urinary bladder (UB). Urethra, vagina (V), and rectum (R) join into a common channel (CC). ( **B** ) After separation of the rectum (R), a vertical incision is made in the posterior wall of the common channel (broken blue line). ( **C** ) The posterior vaginal wall (red line) is displaced downwards to close the posterior defect in the common channel.

Through the open defect in the posterior wall of the vagina/sinus, we can inspect the urogenital confluence from inside and we can now see the catheter running up the common sinus through the urethra into the urinary bladder. Also, we can inspect the inside of the vagina and divide/excise a longitudinal vaginal septum if present ( Fig. 5A ). The defect is then widened distally via a vertical incision (∼1 cm) through the posterior wall of the common urogenital sinus toward but not reaching the perineum ( Fig. 5B ). This vertical defect is then closed horizontally displacing the posterior vaginal wall downwards toward the perineum (posterior sinuplasty) ( Figs. 5C and 6 ).

**Fig. 7:**
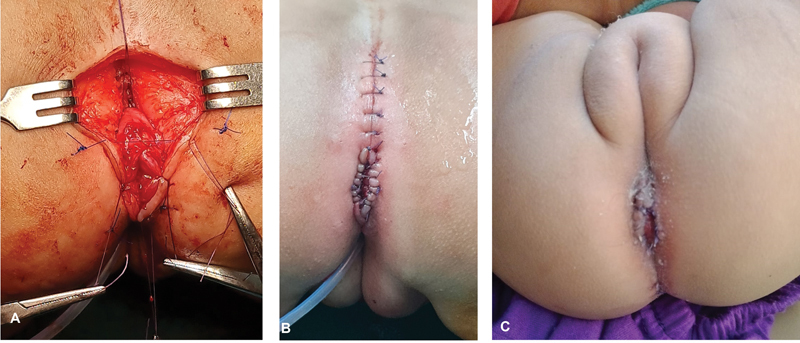
Operative steps of posterior sagittal anorectoplasty (PSARP) (continued). ( **A** ) The pelvic floor and striated sphincter muscles are reconstructed around the neo-anus and rectum. ( **B** ) The anocutaneous anastomosis is performed at the predestined site of the normal anus. ( **C** ) Follow-up after 2 weeks.

Finally, the anorectoplasty is completed by performing the anocutaneous anastomosis at the predestined site of the normal anus, while the pelvic floor and striated sphincter muscles are reconstructed around the neo-anus and rectum ( Fig. 7 ).

The urinary catheter is removed after 7 days. Follow-up ultrasound of the abdomen and pelvis is performed 1 month after operation to check for preoperative hydrocolpos and dilatation of the upper urinary tract. Ultrasound is repeated after 6 months, and then yearly till and through puberty. Calibration of the neo-anus by metal (Hegar) dilators starts 2 to 3 weeks after the anorectoplasty and is continued for 4 to 6 months. Closure of the colostomy is scheduled 3 to 4 months after the anorectoplasty.

Case 1: The first case was referred to our facility from a distant governorate for the definitive repair at the age of 18 months. She had a pelvic colostomy performed at birth without any sort of urogenital decompression despite the presence of associated hydrocolpos. Associated anomalies included right hydroureteronephrosis, ectopic (low position) left kidney, and longitudinal vaginal septum ( Fig. 1 ). There was history of attacks of urinary retention; the urinary bladder was felt and seen distended in preoperative imaging. Cystoscopic examination was performed upon referral, and a urinary catheter was secured inside the bladder to avoid attacks of retention till time of operation, which was scheduled on the nearest operative list. As described before, the anorectoplasty (PSARP) was performed in addition to excision of the longitudinal vaginal septum and posterior sinuplasty. Postoperative recovery was uneventful, the urinary catheter was removed after 7 days, and the patient could void spontaneously. After discharge, the patient returned to her original governorate to complete her management and follow-up there. The parents were recently contacted by phone: the patient is now 3 years old; she underwent closure of colostomy and have started training for urinary and bowel control (voluntary bowel motions). Repeated follow-up ultrasound examinations were essentially normal apart from ectopic left kidney.

Case 2: The second case had a pelvic colostomy performed at birth in addition to tube vaginostomy (via a self-retaining Foley's catheter) for associated hydrocolpos ( Fig. 2 ). PSARP and posterior sinuplasty were performed as described at the age of 7 months with uneventful postoperative recovery. The urinary catheter was removed after 7 days, while the vaginostomy tube was left open for 2 weeks to make sure that there was no urine flow through it after operation. The vaginostomy tube was then clamped and removed leaving its hole in the anterior abdominal wall to close spontaneously. Calibration of the neo-anus started 2 weeks after operation to be continued for 4 to 6 months as usual. Closure of colostomy was performed 3 months later. Ultrasound of the abdomen and pelvis at 1 and 6 months' follow-up were normal (no vaginal distension, no dilatation of the urinary tract). The patient is still below the age of bowel control; however, the parents report spontaneous uncomplicated urination and defecation.

**Fig. 8:**
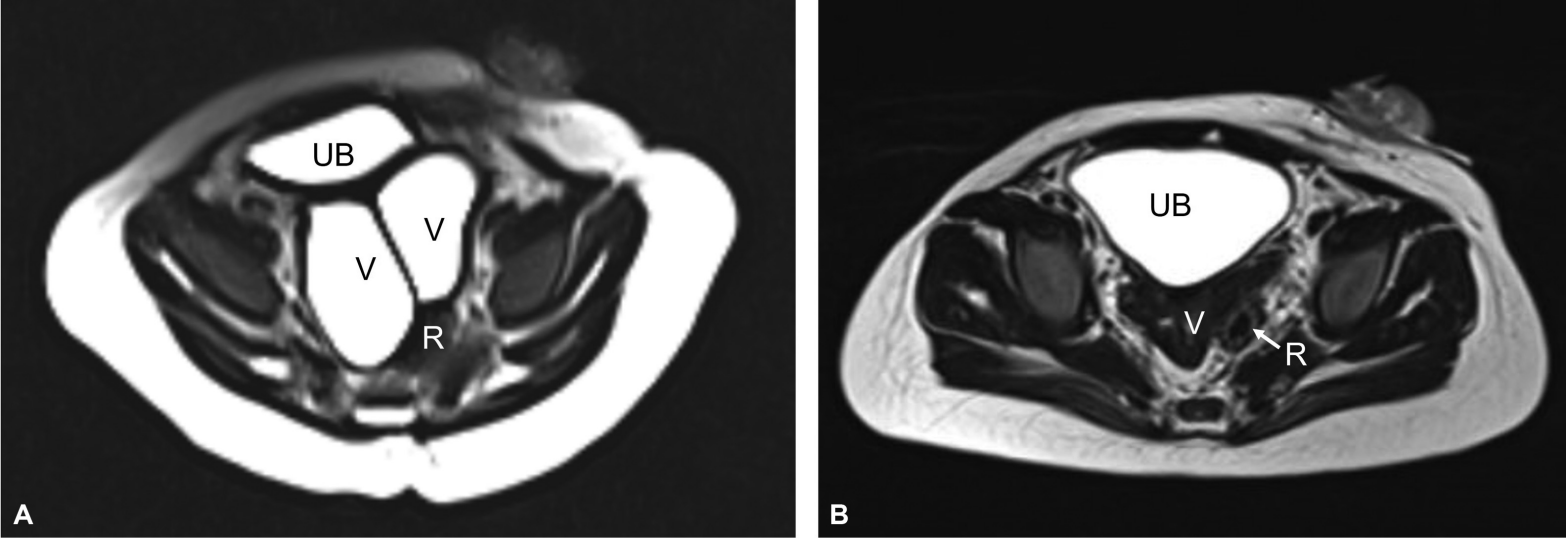
Pelvic magnetic resonance imaging (MRI) (axial T2-weighted image [T2WI]) of the third case comparing vaginal distension before ( **A** ) and after operation ( **B** ). Urinary bladder (UB), vagina (V), and rectum (R). Note vaginal collapse in ( **B** ) after posterior sinuplasty and excision of longitudinal septum.

Case 3: The third case was referred for definitive repair at the age of 6 months. Like the first case, only colostomy was performed at birth without active management for associated hydrocolpos. Preoperative imaging demonstrated bilateral hydroureteronephrosis in addition to markedly distended two hemi-vaginas ( Fig. 3 ). Anorectoplasty (PSARP), excision of vaginal septum, and posterior sinuplasty were performed with uneventful postoperative recovery ( Figs. 4 5 6 7 ). Urinary catheter was removed, and anal calibration was performed as usual. Follow-up postoperative ultrasound of the abdomen and pelvis was normal. Postoperative pelvic MRI (3 months later) confirmed adequate drainage of hydrocolpos ( Fig. 8 ) and good location of neorectum within muscle complex. The case is being prepared to close the colostomy.

## Discussion

Surgical treatment of cloaca represents a serious challenge. 3 6 10 Peña described vaginal separation from the urinary tract as technically difficult and very time consuming. High rate of complications is probably related to devascularization of these fine structures during mobilization. 6 In 1997, Peña introduced a new concept for mobilization of the urethra and the vagina as one unit (total urogenital sinus mobilization), which he described as “an easier way to repair cloaca.” 6 At that time, Hendren commented that “there is never going to be an easy way to perform a cloaca,” 6

Time has passed, and maybe Dr. Hendren's words proved to be true. Despite the reported success of the total urogenital mobilization by Dr. Peña, the results have not always been reproducible at different centers. 2 3 4 11 Also, the long-term consequences of the procedure on urinary continence remain a major concern. 2 3 4 5 A high percentage of cloaca patients (40%) cannot evacuate their bladder spontaneously and depend on clean intermittent catheterization (CIC). 2 3 It is not known exactly whether this is related to “pure” innate factors or maybe related to a second hit (iatrogenic) at operation. 4

In this report, we present a new strategy to manage hydrocolpos and effectively decompress the genital tract in cases of cloaca without the need for major dissection to separate the vagina and urethra at an early age. The surgical technique aims to disrupt the valve mechanism at the level of the urogenital confluence which is responsible for entrapment of urine inside the vagina. 4 8 Simply, a vertical incision is made in the posterior wall of the common urogenital sinus starting from the distal vagina down toward but not reaching the perineum, which is then closed horizontally. This would provide wider drainage and prevent urine entrapment inside the vagina (hydrocolpos). These girls may have the opportunity to complete their staged anorectoplasty during infancy, and with a well-drained genital tract. Meanwhile, we can follow and study their natural voiding pattern (bladder evacuation) without bias and after excluding the potential risk of iatrogenic damage from extensive dissection in this critical area.

The importance of starting early and effective urogenital decompression in cases of cloaca (at time of colostomy) is well established in the literature to avoid possible adverse effects on the upper urinary tract. The latter is the main source of morbidity in cloaca, 1 4 7 which was evident in two out of three cases in this report. Here, we should emphasize that the technique described in this report “posterior sinuplasty” is a delayed procedure performed later during infancy at time of anorectoplasty. In other words, posterior sinuplasty cannot replace early urogenital decompression that should start from day 1 when indicated. There are several ways to start early urogenital decompression in cases of cloaca while waiting for the definitive repair: vaginostomy, vesicostomy, or CIC. Although there is no consensus on the best way to decompress the urogenital tract in cloaca in the neonatal period, yet whatever you do, make sure it is working. 7

Delaying vaginal surgery in cloaca patients after puberty has been a matter of debate. 5 deVries stated that “the choice of time and the extent of definitive correction of associated genital defects should be dictated by the individual's pathology.” Hendren advocated early urogenital correction at the same time of anorectoplasty when exposure is “perhaps” optimum. 12 Others prefer delaying vaginoplasty to early adolescence when the risk–benefit can be better assessed, and a more definitive procedure can be performed. 10 Another potential advantage for delaying vaginal surgery in such cases would be the improved vascularity of the genital tract under effect of postpubertal hormones. 5 We do agree that in simple forms of the anomaly (common sinus ∼1–2 cm), a low vagina can be fully corrected during the anorectoplasty with favorable and reproducible outcomes. 8 However, with more severe forms, we may adopt the latter option of delaying vaginal surgery after providing adequate and permanent drainage for associated hydrocolpos; this will enable us to remove the vaginostomy and close all stomas in infancy after successful anorectoplasty.

Cloaca represents a diverse and complex spectrum of anomalies. A single technique or approach is not expected to fit for all grades of the disease. This report is concerned with intermediate forms of cloaca associated with hydrocolpos that can be approached through the perineum. The distal rectum is usually opposite mid-sacrum (S3) or below; the distal vagina is opposite the mid-pubis with a common channel 2 to 3 cm long. 7 Other types of cloacae with longer common channel and higher rectum will need abdominal approach. 7 This report included only three cases and the follow-up period is still short. However, our preliminary results of posterior sinuplasty show clear radiological evidence for successful genital decompression. Continued follow-up is essential to assess continence in these girls and the long-term effect on upper urinary tract.

## Conclusion

In selected cases of cloaca, posterior sinuplasty is a simple procedure that can be applied during anorectoplasty to provide effective drainage of associated hydrocolpos. Posterior sinuplasty would allow reversal of higher genital decompression (vaginostomy) performed at the time of colostomy.
